# Variation in alarm calls during different breeding stages of the common kestrel (*Falco tinnunculus*)

**DOI:** 10.1242/bio.056648

**Published:** 2021-01-11

**Authors:** Xiaona Huo, Lei Zhou, Jiang Feng, Hui Wu

**Affiliations:** 1 College of Life Science, Jilin Agricultural University, Changchun 130118, China; 2 College of Animal Science and Technology, Jilin Agricultural University, Changchun 130118, China

**Keywords:** Acoustic signals, Alarm calls, Breeding stages, Common kestrel

## Abstract

Acoustic signals play a key role in animal communication. Animals usually use alarm signals to warn mates or offspring of the presence of threats or to intimidate or distract predators. Birds commonly use acoustic signals as a means of communication. Alarm calls in passerines at different breeding stages can reflect their nest defense intensity. However, little is known about the characteristics, plasticity, and impact factors of alarm calls during the reproductive period in raptors. Here, from March to July in 2019, the alarm calls of eight pairs of common kestrels (*Falco tinnunculus*) during the breeding period were recorded using a portable recorder with a strongly directed microphone in the Zuojia Nature Reserve, Jilin province, China. The differences in acoustic parameters of parental alarm calls in different breeding stages were analyzed. The results showed that the alarm calls of common kestrels were composed of multi-harmonic arched frequency modulation with the maximum energy distribution in the second harmonic. The duration and rate of the alarm calls increased significantly as the breeding season progressed, showing that parents spent increasing amounts of time on nest defense. Additionally, the acoustic parameters of alarm calls in common kestrels were significantly different depending on offspring numbers, suggesting that offspring numbers influenced parental nest defense. These results showed that differences in alarm calls during different breeding stages may reflect a trade-off between defense costs and reproductive benefits.

## Abstract (precis)

**Summary:** Our results clearly showed parental alarm calls varied as the breeding cycle progressed in the common kestrel, and further suggested offspring numbers influenced parental nest defense.

## INTRODUCTION

Acoustic signals play a key role in animal communication, such as in mate selection, resource defense, and individual or species recognition (Marler and Slabbekoorn, 2004; Wilkins et al., 2013). Acoustic signals are widely used for communication in birds (Mckown, 2008), and in the other animals, such as insects, frogs, bats, cetaceans, and primates (Ovaska and Caldbeck, 1997; Fedurek and Slocombe, 2011; Lin et al., 2016; Natasha et al., 2017; Schevill and Mcbride, 1956). Many birds and mammals produce alarm signals to warn of danger or the presence of predators (Caro and Girling, 2005; Fasanella and Fernández, 2009). Such signals are directed towards mates (Greig-Smith, 1980; Morton and Shalter, 1977), offspring (Platzen and Magrath, 2004, 2005), or the predators themselves (Greig-Smith, 1980). In response to predators, birds use alarm calls to inform and deter predators (Courter and Ritchison, 2010; Greig-Smith, 1980), or to prompt nestlings to adopt anti-predation behaviors (McIntyre et al., 2014). Alarm calls in some bird species convey rich information about the type and size of the predators or the level of threat (Shah et al., 2015; Suzuki, 2014; Templeton et al., 2005). Some birds can respond to predators by producing different types of calls, by varying the rate, frequency, and number of calls (Courter and Ritchison, 2010; Soard and Ritchison, 2009; Templeton et al., 2005). Thus, alarm calls can provide crucial information in high-risk situations and have been shown to reduce the likelihood of birds being killed by a predator (Griesser, 2013).

Parental alarm calls can be considered a form of defense strategy to prevent predators from approaching nests (Caro and Girling, 2005). This behavior not only affects survival but also increases reproductive success in many birds (Galeotti et al., 2000). However, production of alarm calls incurs a loss of time and energy. As a result, less time and energy can be allocated to hunting, which may decrease the future reproductivity of parents. Additionally, producing alarm calls may exposes their position, which may increase the probability of injury or death to the parent (Fasanella and Fernández, 2009; Wallin, 1987; Brunton, 1986). Therefore, parents may adjust their investment in the production of alarm calls during different breeding stages by making a trade-off between the costs and benefits in nest defense. In this case, it may be beneficial for parents' survival and future reproduction. Parental defense behavior is used as a measure of risk taking, and thus, parental investment (Trivers, 1972). According to the parental investment theory (Trivers, 1972; Dawkins and Carlisle, 1976; Klug et al., 2012), parents weigh the costs and benefits of investment based on the value of their offspring and the value of future reproduction. Parental defense investment varies depending on the value of eggs and nestlings to adults. In this case, alarm calls, which are a form of parental nest defense, may increase with the growth of nestlings because the reproductive value of nestlings increases with age (the ‘reproductive value hypothesis’) (Redondo and Carranza, 1989; Fasanella and Fernández, 2009) or as nestlings become visually or acoustically conspicuous to predators (the ‘vulnerability hypothesis’) (Andersson et al., 1980; Greig-Smith, 1980; Weatherhead, 1979; Kleindorfer et al., 1996). These two hypotheses explain that parental alarm calls increase with the age of offspring in altricial birds (Kleindorfer et al., 1996; Redondo and Carranza, 1989). Thus, the parental decision to produce alarm calls depends on multiple factors such as parental investment, nestling age, offspring number (clutch/brood size), laying date, sensitivity to predators, and future reproductive value (Buraer et al., 1989; Fasanella and Fernández, 2009; Caro and Girling, 2005).

So far, most studies have focused on the types, structure, and functions of alarm calls in passerines (Cunningham and Magrath, 2017; Randler, 2013; Suzuki, 2011; 2014). Little is known about the characteristics, plasticity, and impact factors of alarm calls during the reproductive period in non-passerines, especially in raptors. Raptors are relatively longer-lived than passerines and are capable of attacking and harming potential predators (Galeotti et al., 2000). Although raptors have less well-developed vocalizations than passerines, their vocalizations are complex, and some raptors also use alarm calls to defend their nests (Kumar et al., 2018; Kuca and Yosef, 2016). Some studies have indicated that a few non-passerines vary alarm calls to convey information about predators. For example, domestic chickens (*Gallus domesticus*) varied the characteristics of their alarm calls to convey information about perceived threats (Wilson and Evans, 2012). However, little is known about the features and functions of the alarm calls in raptors during different breeding stages. Thus, it is necessary to understand the trade-off between antipredator investment and reproductive investment in different breeding periods and determine whether raptor parental alarm calls change with the value of offspring and the value of future reproduction.

The common kestrel (*Falco tinnunculus*) is a medium-sized raptor belonging to the Falconidae. It is highly territorial and often disturbed by intruders (including humans) in the breeding season. We found that common kestrels usually emit alarm calls to defend their nests when potential predators or intruders, including humans, entered their nest territories during our previous field investigation. Most birds, including raptors, treat humans as potential predators and are sensitive to human proximity (Beale and Monaghan, 2004; Newton, 2011). Therefore, in this study, we investigated the alarm calls of common kestrels and the factors that affected alarm calls during different breeding stages. The goals of this study were (1) to describe the characteristics of alarm calls in common kestrels; (2) to examine how parental alarm calls vary with the growth of offspring during different breeding stages; and (3) to assess whether offspring number affects parental alarm calls.

## RESULTS

### The characteristics of alarm calls during the breeding period

**Fig. 1.**
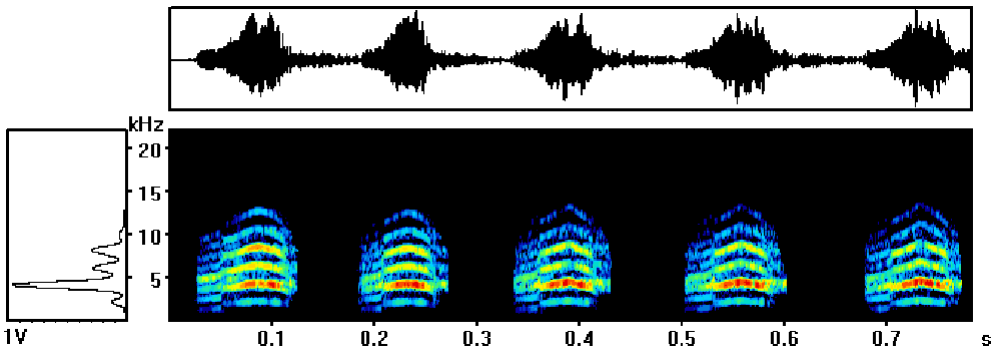
**The spectrogram (center), corresponding oscillogram (top) and power spectrum (left) of alarm calls of a randomly chosen common kestrel.**

In this study, the alarm calls of common kestrels were characterized by loud, wide-band calls and were composed of multi-harmonic arched frequency modulation (CFM) with the distribution of the maximum energy in the second harmonic (Fig. 1). The frequency range was 1.2–20.0 kHz, and the peak frequency was between 3.2 and 4.4 kHz. The duration of the calls ranged from 0.429 s to 5.436 s.

### Variation of alarm calls in the different breeding stages

**Fig. 2.**
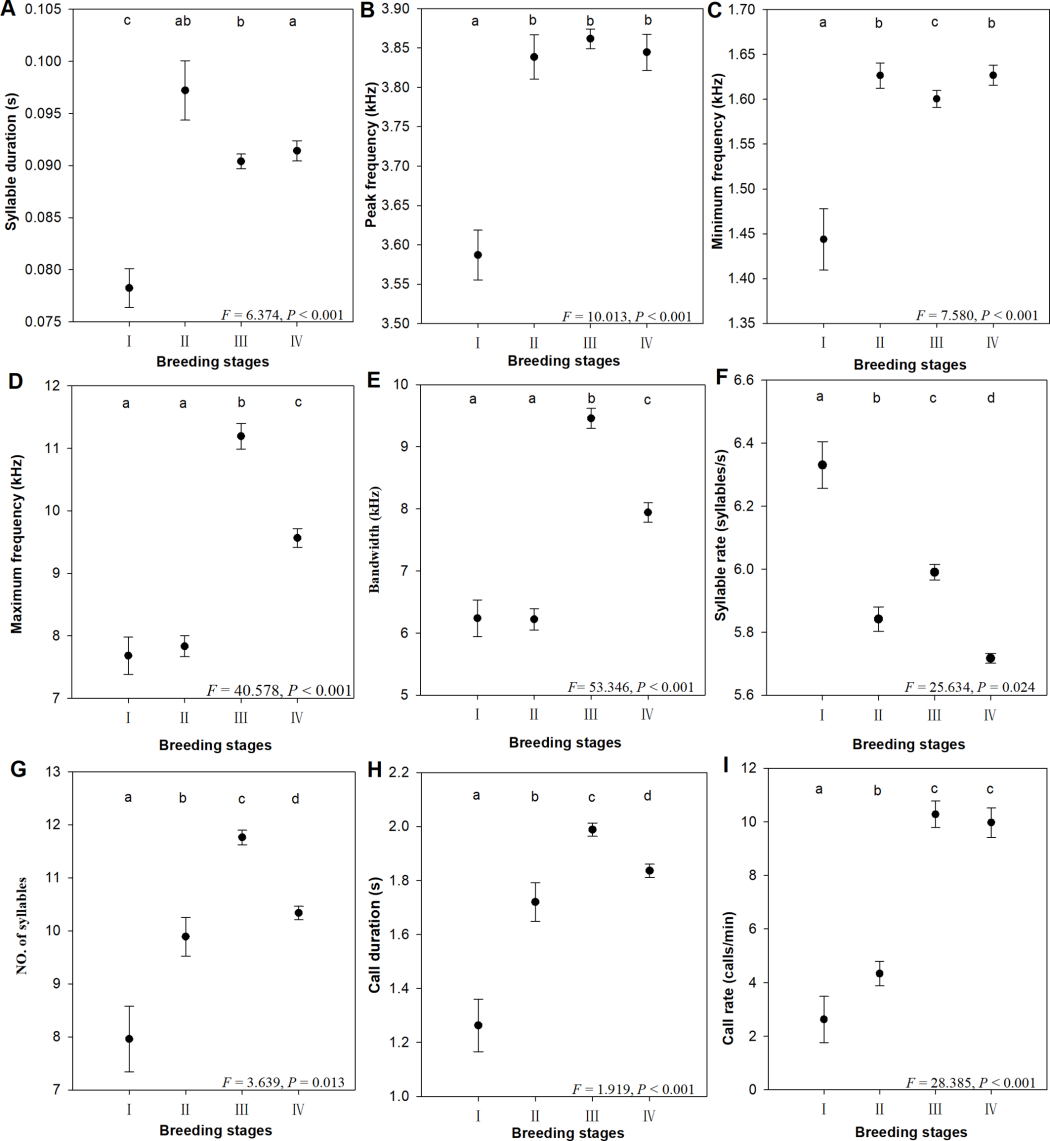
**Stage variation in call variables of alarm calls of *F. tinnunculus* during breeding period.** I: incubation stage; II: early-nestling stage; III: second-nestling stage; IV: late-nestling stage. A: syllable duration; B: peak frequency; C: minimum frequency; D: maximum frequency; E: bandwidth; F: syllable rate; G: number of syllables; H: call duration; I: call rate.

All acoustic parameters of alarm calls differed significantly in different breeding stages (*F*>1.92, *P*<0.001; Table 1). Compared with the incubation stage, significant increases in syllable duration, peak frequency, minimum frequency, maximum frequency, and bandwidth in three nestling stages were observed, which means that values of the five parameters were lowest during the incubation stage (Fig. 2A–E). However, the syllable rate decreased during the breeding period (Fig. 2F). The number of syllables and call duration gradually increased from the incubation stage to the nestling stages (Fig. 2G–H). During the breeding period, the call rate increased as the breeding season progressed (*F*=28.385, *P*<0.001; Fig. 2I).

**Table 1.**
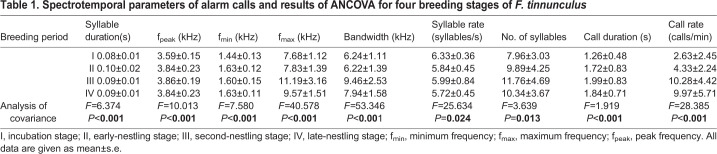
**Spectrotemporal parameters of alarm calls and results of ANCOVA for four breeding stages of *F. tinnunculus***

**Fig. 3.**
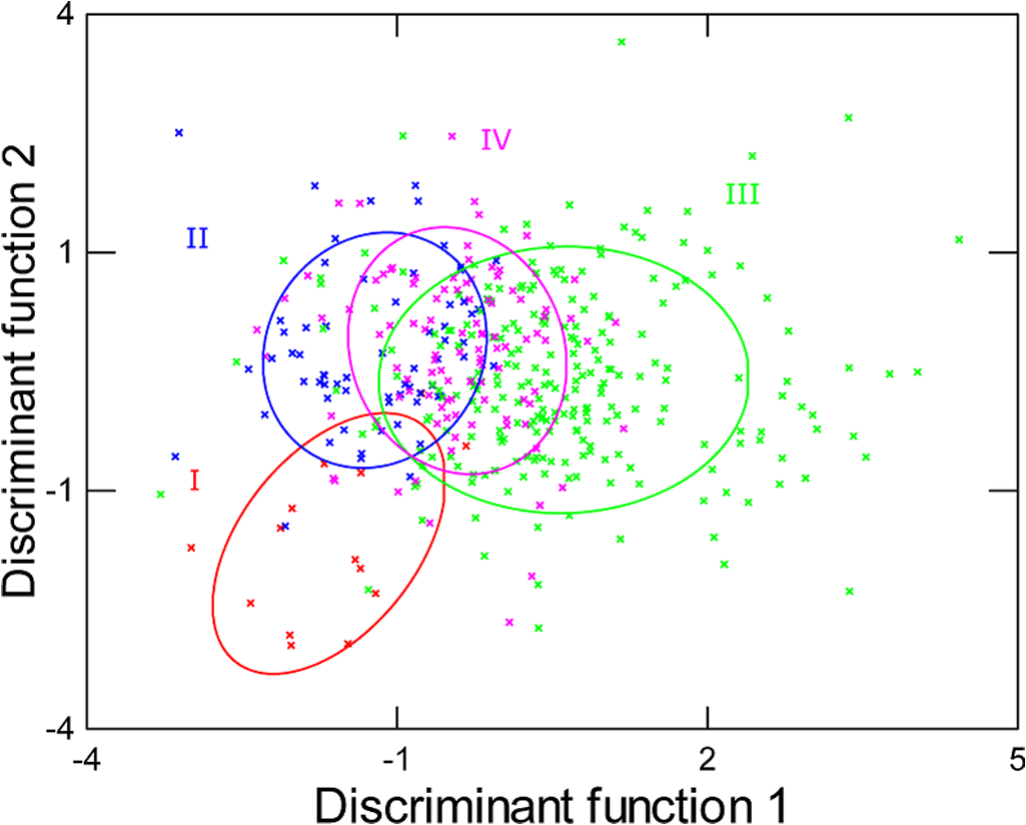
**Ellipses** **showing the 50% confidence interval obtained from a discriminant function analyze of five principal component factor scores measured from alarm calls.** Colors show grouping according to breeding stage (*n*=4).

We extracted five principal components (PCs) (with eigenvalues >1), which explained 81.981% of the total variance (Table 2). PC1 explained 23.549% of the variance, PC2 explained 21.398% of the variance, PC3 explained 13.002% of the variance, PC4 explained 12.220% of the variance, and PC5 explained 11.812% of the variance. PC1 was related to the number of syllables and call duration. PC2 was related to maximum frequency and bandwidth. PC3 was related to peak frequency and minimum frequency. PC4 was related to syllable rate and call rate. PC5 was related to syllable duration (Table 2). A linear discriminant function analysis showed that 66.7% of the alarm calls were correctly assigned to their own stages (Table 3; Fig. 3). The percent of correctly classified calls was significantly higher than correct classification by chance (chance level: 25%; two-tailed binomial test: *P*<0.001).

**Table 2.**
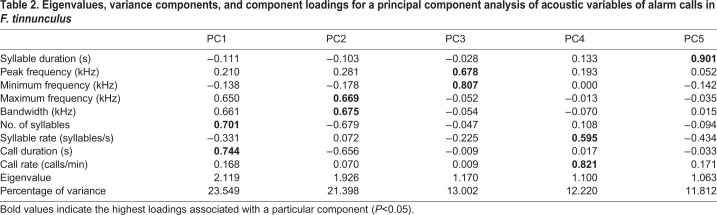
**Eigenvalues, variance components, and component loadings for a principal component analysis of acoustic variables of alarm calls in *F. tinnunculus***

**Table 3.**
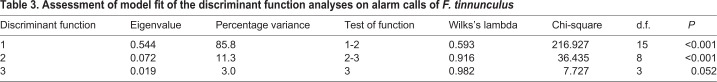
**Assessment of model fit of the discriminant function analyses on alarm calls of *F. tinnunculus***

### The effects of offspring numbers on alarm calls

**Fig. 4.**
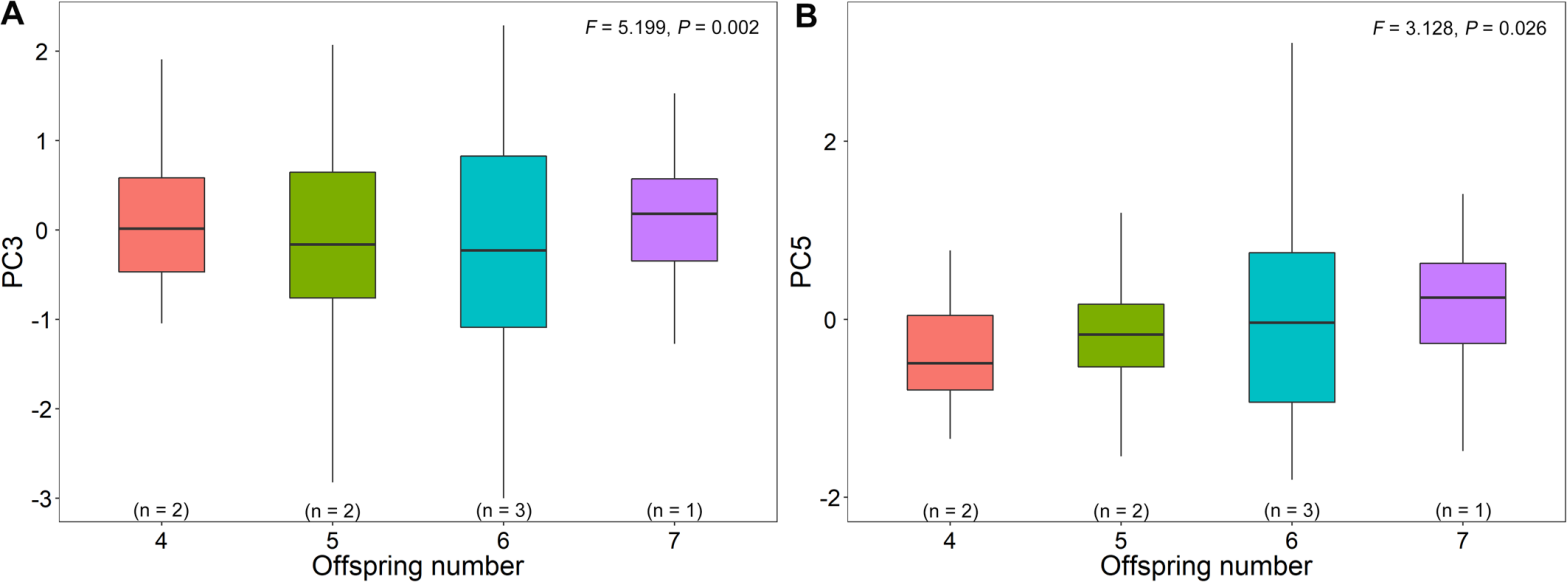
**The relationship between PC3** **(A)** **and PC5 (B) of acoustic parameters of alarm calls in breeding period and offspring number (eggs and/or nestlings).**

We extracted five components from nine acoustic parameters of alarm calls during the breeding period, including the incubation stage and nestling stages, which explained 81.981% of the total variance (Table 2). Offspring number had a significant effect on PC3 and PC5 from alarm call parameters (*F=*5.199, *P=*0.002; *F=*3.128, *P=*0.029), while breeding stage had a significant effect on PC5 from alarm call parameters (*F=*4.820, *P=*0.029; Table 4). PC5 (relating to syllable duration) increased along with offspring number, while PC3 (relating to peak frequency and minimum frequency) was lowest when there were six offspring (Fig. 4).

**Table 4.**
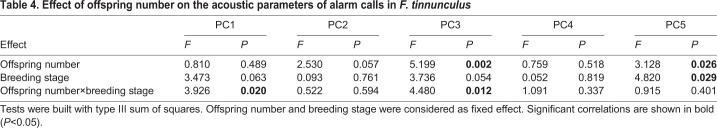
**Effect of offspring number on the acoustic parameters of alarm calls in *F. tinnunculus***

## DISCUSSION

Like most birds, the common kestrel uses alarm calls to defend its nest in the breeding season. The alarm calls of common kestrels were composed of several CFM syllables. The most energy was distributed in the second harmonic. Alarm calls were the most common vocal response in nest defense when encountering predators (humans). Our results showed that the alarm calls varied among breeding stages, suggesting that parental investment changed in different breeding stages. We also found that the offspring number in common kestrels affected their alarm calls during the breeding season.

Common kestrels emitted alarm calls when they encountered intruders or predators. Such calls in passerines and other birds signal the approach or presence of both airborne and terrestrial potential predators (Brown and Veltman, 1987; Klump and Curio, 1983; Marler and Slabbekoorn, 2004; Morton and Shalter, 1977). For example, alarm calls are used as a form of response to predators or intrusion by humans in the white-tailed hawk (*Buteo albicaudatus*) and northern goshawk (*Accipiter gentilis*) (Farquhar, 1993; Kennedy and Stahlecker, 1993). The alarm calls of some raptors are similar in call structure each other. For example, the Australian hobby (*Falco longipennis*) has similar alarm calls to the Australian kestrel (*Falco cenchroides*) (Hollands, 1984). The alarm calls of the peregrine falcon (*Falco peregrinus*) and black falcon (*Falco subniger*) are similar, and the calls of Australian hobbies given when birds were alarmed appeared to be shorter, higher-pitched variations of peregrine falcon calls (Jurisevic, 2016). These raptors emitted harsh and/or harmonic wide-band calls when alarmed, which was consistent with our study.

The alarm calling rate may be a measure of nest defense intensity (Bjerke et al., 1985). The call rate of alarm calls in common kestrels increased during the nestling season in our study. This result was in agreement with the predictions of both the brood value hypothesis (Montgomerie and Weatherhead, 1988) and the vulnerability hypothesis (Andersson et al., 1980; Buraer et al., 1989; Weatherhead, 1979). These hypotheses were derived from the parental investment theory, which would predict that parental alarm calls increase with the age of offspring. Variation in alarm calls indicates differences of parental investment to some extent during different breeding stages. For example, defense intensity in the black kite (*Milvus migrans*) increased as the breeding cycle progressed, suggesting that parents tuned their defense response in relation to their parental investment (Kumar et al., 2018). The reason why defense investment varies in different breeding stages may be that the relative importance of offspring compared to the future fitness of parents increases over time from egg-laying to the fledging of young (Cavalli et al., 2017). The kestrel called more frequently during the nestling period than during the incubation period, that is, the call rate increased. Interestingly, we did not find an increase in syllable rate during the breeding period, which may be because an increase in call rate may lead to less energy allocated to the syllable rate. Thus, the common kestrel makes a trade-off between increasing call rate and increasing syllable rate because both adjustments have a high energy cost. Parents should be selected to maximize the benefits of alarm calls with decreasing risks (Montgomerie and Weatherhead, 1988; Redondo and Carranza, 1989). The risk that the parents take to defend their offspring could thus be expected to increase with the age of their offspring (Andersson et al., 1980). This hypothesis was supported by our results, which showed that common kestrels exhibited little or no nest defense and fewer alarm calls during the incubation period. Similarly, McKell and Gary (1993) found no evidence of nest defense by eastern screech-owls (*Otus asio*) during the incubation period. Situations in which the defense investment for nestlings is more than the investment for eggs have been observed in various passerine birds (Burhans, 2001; D'Orazio and Neudorf, 2008; Patterson et al., 1980; Redmond et al., 2009). After the incubation period, the duration of common kestrel parental alarm calls increased during the nestling period. In the same way, the red-backed shrike (*Lanius collurio*) produced more alarm calls when their brood was in the nestling stage than in the incubation stage (Strnadová et al., 2018). Since parents have limited energy, nest defense may result in the loss of future reproductive success due to time and energy costs, injury, or death (Galeotti et al., 2000). In this case, the common kestrel may be more willing to defend nestlings compared to eggs. Thus, it is common that parents defending their nests actively adjust their defensive behavior according to the cost/benefit trade-off (Andersson et al., 1980; Dugatkin and Godin, 1992; Mahr et al., 2015). Although Gill and Sealy (2004) found that female yellow warblers (*Dendroica petechia*) gave more alarm calls with longer duration when laying than when caring for nestlings, this may have been because their eggs were likely to be parasitized during the incubation period. To sum up, it is rational in most birds to display more parental investment during the nestling period than during the incubation period. To some extent, the variation of alarm calls reflects parental investment in nest defense.

Life history theory can be used to explain how energy is distributed between growth, maintenance, and reproduction (Stearns, 2000). Because the energy available to an organism is finite, the investment of parents in their offspring is limited, and the fitness value is significant for parental survival (Eric and John, 1974; Wimsatt, 1970). Thus, the number of offspring in the nest may affect the fitness of the parents. Indeed, in this study, offspring number had an effect on the acoustical features of alarm calls. These results indicated that defensive investment in common kestrels varied with the offspring value. Several studies have reported positive links between clutch size and parental nest defense (Caro and Girling, 2005; Curio et al., 1984; Montgomerie and Weatherhead, 1988). Since parental investment in nest defense could be related to the value of current offspring (Curio, 1987), parents invested more in defense when there were more eggs or nestlings, consistent with the hypothesis that parental nest defense should increase with the reproductive value of the offspring (Curio and Regelmann, 1987; Kozma and Kroll, 2010; Montgomerie and Weatherhead, 1988; Svagelj et al., 2012; Wiklund, 1990). Newton argued that the larger the broods, the greater the fitness gains for adults (Taylor and Newton, 1990). It is likely that the greater the offspring number, the more parents are willing to invest in defense. Our results were consistent with nest defense reported in red-winged blackbirds (*Agelaius phoeniceus*) (Robertson and Biermann, 2015), tawny owls (*Strix aluco*) (Wallin, 1987), and merlins (*Falco columbarius*) (Wiklund, 1990). However, some studies have shown that there was no relationship between nest defense and fitness values (Hakkarainen and Korpimäki, 1994; Redmond et al., 2009; Tryjanowski and Golawski, 2004). It may be that defense investment is influenced by other factors such as parental age, experience, and threats in the environment. Future studies should consider the effect of these factors on alarm calls in nest defense in order to better study the variation of calls.

In conclusion, our study showed that common kestrels produced loud, wide-band alarm calls composed of several CFM syllables when encountering humans in their nest territory. The parental nest defense increased with the reproductive value of the nest contents (eggs or nestlings), which may be the results of a trade-off between defense costs and reproductive benefits. Our results improved our understanding of the plasticity and function of raptor alarm calls and how varied alarm calls convey information about the environment to raptors' mates or offspring. A limitation of our study was that the sample size was relatively small because most common kestrels in the area use natural nests rather than artificial nest-boxes, which makes it difficult to observe and record alarm calls. Future studies should investigate more pairs of common kestrels using advanced automated sound and behavior monitoring technology.

## MATERIALS AND METHODS

### Study area and species

The study was conducted in the Zuojia Nature Reserve (126°00′-126°09′E, 44°01′-44°06′N) in Jilin Province, a mountainous secondary forest located in northeastern China. The study area comprises different types of vegetation such as shrubs, arbors, meadows, and herbs. Local predators include the brown wood-owl (*Strix uralensis*) and the collared sparrowhawk (*Accipiter cirrocephalus*). The common kestrel is a small diurnal raptor that breeds in old stick-nests (mainly magpie) and nest boxes. The incubation period of common kestrels is 28–32 days long and the nestling period is at least 30 days long. Females start incubating their eggs before the clutch is complete, creating an asynchronous hatching pattern within the brood (Martínez-Padilla and Viñuela, 2011). The first two or three nestlings within a brood are the same age (personal observation). There are distinct differences between the sexes in the breeding duties of common kestrels. Males hunt and provide food for the females and the nestlings, while females incubate the eggs, brood the offspring, and divide the food for them for one and half to two weeks after hatching. From 1.5–2 weeks of age, nestlings need to be fed by parents, and after 2 weeks, the nestlings can feed on their own. The nestlings begin to fledge at about 30 days old. Common kestrels frequently produce alarm calls for nest defense during the breeding period when encountering predators in our study area.

### Data collection

All nest-boxes were monitored regularly by climbing the nest tree from March to July in 2019 to determine the laying date, clutch size, and brood size. We monitored eight nests during the breeding season. All nests (*N*=8) were tested only once at each stage. To examine the variation of alarm calls during the breeding season, the breeding cycle was divided into four stages: (1) incubation stage: from the time the first egg was found until the first nestling was hatched; (2) early-nestling stage: when the first nestling was 0–10 days old; (3) second-nestling stage: when the nestling was 11–20 days old; and (4) late-nestling stage, when the nestling was 21–30 days old.

An experimenter walked directly towards the nest tree at a slow, constant speed (approximately 0.5 m/s). An observer who standing at least 30 m away from the nest tree observed the behavior of the adult birds. Since we mainly compared the variation of alarm calls in nest defense with breeding stages, we only recorded the calls. A TASCAM HD-P2 digital recorder (TEAC Corporation, Tokyo, Japan) and a Sennheiser MKH416 P48 microphone (Sennheiser electronic GmbH and Co. KG, Wedemark, Germany) were set up 10 m away from the nest to record the alarm calls of the common kestrel. The sampling frequency of calls was set at 44.1 kHz with 16 bits. Meanwhile, we recorded the current breeding stage and clutch or brood size. There were two nests in which five eggs were laid and four nestlings were hatched.

### Alarm calls analysis

Avisoft SAS Lab Pro 5.1.09 software (Avisoft Bioacoustics, Berlin, Germany) was used to analyze the acoustic parameters of alarm calls during the breeding period. We only analyzed the first 3 min of each vocal response at every stage. From these recordings, we selected high-quality calls without overlap with noise from wind and other sources to determine call parameters. Before analysis, the calls were high-pass filtered at 1 kHz to remove background noise. In total, 413 alarm calls were used for the acoustic analyses, including 14 calls (*n*=5) in the incubation stage, 66 calls (*n*=5) in the early-nestling stage, 240 calls (*n*=6) in the second-nestling stage, and 101 calls (*n*=5) in the late-nestling stage. We defined a syllable as the continuous sound, and a call composed of one or several syllables in a whole vocalization. For each acoustic analysis we chose a set of nine acoustic parameters that broadly described the temporal and spectral characteristics of the alarm calls. We obtained acoustic measurements from spectrograms using a 1024-point fast Fourier transform with 75% overlap. Nine parameters were measured including syllable duration (s), minimum frequency (kHz), maximum frequency (kHz), peak frequency (kHz), bandwidth (kHz), syllable rate (number of syllables per second for each call; syllables/s), number of syllables, call duration (s), and call rate (number of calls per minute; calls/min) (see Table 1).

### Statistical analysis

We assessed the normality of the data using Kolmogorov–Smirnov (K-S) tests. An analysis of covariance (ANCOVA) was conducted to assess the difference between different breeding stages with acoustic parameters as the dependent variable and the ID of individuals as the univariate. To avoid pseudo-replication, we used individual mean values of every acoustic parameter for the breeding stage analysis.

Once significant differences were found, differences among each stage were tested using specific post-hoc tests. To determine whether alarm calls could be correctly assigned to the breeding stages, we performed a PC analysis on nine acoustic parameters. The PC scores were used to represent alarm calls for the subsequent discriminant function analysis (DFA). We extracted five PCs (with eigenvalues >1), which explained 81.981% of the total variance (Table 2). Subsequently, we performed the DFA analysis and conducted a two-tailed binomial test to determine whether the observed percentage of correct classification was higher than the percentage of random classification.

To examine the effects of offspring number on alarm calls, we used general linear models to determine whether offspring number could predict the acoustic parameters of the calls. In the model, the PCA scores were considered predictive variables, offspring numbers and breeding stages were included as fixed factors, and the ID of individuals were included as random factors. Values were reported as means±s.e. and all analyses were conducted in IBM SPSS Statistics Version 21 (IBM Corporation, Armonk, NY, USA).
